# Global Epidemiology of Gaucher Disease: an Updated Systematic Review and Meta-analysis

**DOI:** 10.1097/MPH.0000000000002506

**Published:** 2022-07-22

**Authors:** Meimei Wang, Fengqin Li, Jing Zhang, Cheng Lu, Weijing Kong

**Affiliations:** * NHC Key Laboratory of Food Safety Risk Assessment, China National Center for Food Safety Risk Assessment; † Department of Pediatrics, Beijing Friendship Hospital, Capital Medical University; ‡ Beijing Hong Jian Medical Device Company, Beijing, China

**Keywords:** Gaucher disease, prevalence, birth prevalence, systematic review, meta-analysis

## Abstract

Gaucher disease [GD], an autosomal recessive lysosomal storage disorder, is characterized by progressive lysosomal storage of glucocerebroside in macrophages predominantly in bone, bone marrow, liver, and spleen. Meta-analysis of global GD epidemiology was not available before this study.

**Methods:**

To provide a systematic review and meta-analysis of birth prevalence and prevalence of GD in multiple countries. MEDLINE and EMBASE databases were searched for original research articles on the epidemiology of GD from inception until July 21, 2021. Meta-analysis, adopting a random-effects logistic model, was performed to estimate the birth prevalence and prevalence of GD.

**Results:**

Eighteen studies that were screened of 1874 records were included for data extraction. The studies that fulfilled the criteria for inclusion involved 15 areas/countries. The global birth prevalence of GD was 1.5 cases [95% confidence interval: 1.0 to 2.0] per 100,000 live births. The global prevalence of GD was 0.9 cases [95% confidence interval: 0.7 to 1.1] per 100,000 inhabitants.

**Conclusions:**

This is the first comprehensive systematic review that presented quantitative data of GD global epidemiology. Quantitative data on global epidemiology of GD could be the fundamental to evaluate the global efforts on building a better world for GD patients.

Gaucher disease [GD], an autosomal recessive lysosomal storage disorder, is characterized by progressive lysosomal storage of glucocerebroside in macrophages predominantly in bone, bone marrow, liver, and spleen.^1^ There are 3 subtypes of GD, which are mostly caused by pathogenic mutation of gene for glucocerebrosidase.^2^ Very rarely, deficiency in the GCase activator, saposin C, could cause GD.^3^

On the basis of pathogenic mechanisms, there are 2 specific ways to treat GD: (1) recovery of enzyme activity, such as enzyme replacement therapy; (2) reduction of accumulation of glucocerebroside in lysosome, such as substrate reduction therapy.^4^ For now, there are several treatments for GD that have been approved, such as Cerezyme^®^ [Genzyme, Cambridge/USA] and Vpriv^®^ [Takeda, Lexington/USA].^1^

There was only one comprehensive review about GD epidemiology.^5^ Nalysnyk et al presented that standardized birth prevalence of GD in the general population varied from 0.39 cases to 5.80 cases per 100,000 live births, and prevalence ranged from 0.70 cases to 1.75 cases per 100,000 inhabitants, respectively.^5^ This study demonstrated more precise results by updating the latest data and presenting quantitative epidemiological data of GD.

## METHODS

### Literature Search Strategy

Preferred Reporting Items for Systematic Reviews and Meta-Analyses (PRISMA) statement was the guideline for this systematic review and meta-analysis.^6^ The complete checklist could be found in Additional file 1. The study strategy adopted to identify studies was as follows:

EMBASE and MEDLINE were searched by terms [“incidence”, “prevalence”, “epidemiolog*” and Gaucher disease”] from inception until July 21, 2021. Endnote X7 was used to manage citations. Detailed literature search strategy for different databases was provided in Additional file 2.

### Inclusion and Exclusion Criteria

Studies fulfilled all of the following criteria were selected: (1) the case collection was based on a field survey; (2) the study was based on population samples rather than volunteers; (3) the study had definite numerator [number of patients] and denominator [number of live births or inhabitants].

Studies that fulfilled any of the following criteria were excluded: (1) study without information available for meta-analysis was excluded; (2) conference abstract was excluded; (3) study that focused on one specific population from one area/country was excluded.

### Quality of Studies

Quality of studies was assessed independently by two reviewers [M.W. and F.L.] based on a checklist specifically for observational studies concerning rare diseases epidemiology, which was adapted from STrengthening the Reporting of OBservational studies in Epidemiology [STROBE].^7^,^8^ Details about this checklist were shown in Additional file 3.

### Data Analysis

Data extraction was operated independently by 2 reviewers [M.W. and F.L.]. For each included study, birth prevalence/prevalence per 10,000 individuals was considered as the primary outcome for meta-analysis. Stata/SE version 15.1 software [StataCorp LP, College Station, TX] was used to conduct statistical analysis. Heterogeneity of epidemiological estimates, along with its derived measure of inconsistency [*I* ^2^], was assessed by Cochran’s *Q* test.^9^ When *P*<0.1 for the *Q* test or *I* ^2^>50%, the signs for substantial heterogeneity, were received, random-effects model was used; otherwise, fixed-effects model was performed. In addition, funnel plot was used to describe potential publication bias.

## RESULTS

**FIGURE 1:**
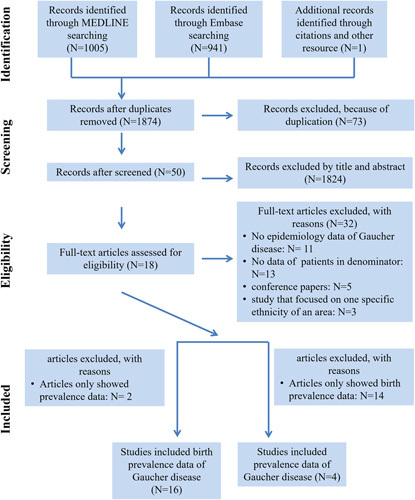
PRISMA flow-chart showing the process of literature search and study selection.

Figure 1 showed the process of identifying eligible epidemiological studies. Eighteen studies, all of which met inclusion criteria and were not excluded by exclusion criteria, were selected and then subjected to quality assessment. Five [28%] studies were rated as high quality, 9 [50%] studies were considered to be of medium quality, and 4 [22%] studies were assessed as low quality (Table 1). Details about the quality of each included study were reported in Additional file 3.

**TABLE 1:** Quality Reporting of Included Studies

References	Was There an Adequate Description of Study Design and Setting?	Was There an Adequate Description of Eligibility Criteria?	Is the Study Population Representative of the Target Population?	Is There an Adequate Description of outcomes?	Is There an Adequate Description of the Study Participants?	Overall Assessment
Meikle et al^10^	Yes	Unclear	Yes	Yes	Yes	Medium
Poorthuis et al^11^	Yes	Yes	Yes	Yes	Yes	High
Applegarth et al^12^	Unclear	Unclear	Yes	Yes	No	Low
Dionisi-Vici et al^13^	Yes	Yes	Yes	Yes	Yes	High
Asuman Ozkara and Topcu^14^	Yes	Unclear	Yes	Yes	Yes	Medium
Revest et al^15^	Yes	Unclear	Unclear	Unclear	Yes	Low
Poupetova et al^16^	Yes	Yes	Yes	Yes	Yes	High
Giraldo et al^17^	Yes	Yes	Yes	Yes	Yes	High
Mechtler et al^18^	Yes	Unclear	Yes	Yes	Yes	Medium
Stirnemann et al^19^	Yes	Yes	Yes	Yes	Yes	High
Liao et al^20^	Unclear	Unclear	Yes	Yes	Yes	Medium
Hult et al^21^	Yes	Unclear	Unclear	Yes	Yes	Medium
Hopkins et al^22^	Yes	Unclear	Unclear	Yes	Yes	Medium
Burton et al^23^	Unclear	Unclear	Unclear	Yes	Yes	Low
Kang et al^24^	Unclear	Unclear	Unclear	Unclear	Yes	Low
Hopkins et al^25^	Yes	Unclear	Unclear	Yes	Yes	Medium
Burlina et al^26^	Yes	Unclear	Unclear	Yes	Yes	Medium
Chien et al^27^	Yes	Unclear	No	Yes	Yes	Medium

More details of quality assessment could be found in Additional file 3.

The studies that fulfilled the criteria for inclusion involved 15 areas/countries. Table 2 showed characteristics of each study. As shown in the table, 10 [56%], 4 [22%], 3 [17%], and 1 [5%] studies were from Europe, North America, Asia, and Oceania, respectively. Primary results showed *P*<0.1 for the *Q* test or *I* ^2^>50%, so random-effects model was used. Variables from outcome measures were pooled using DerSimonian and Laird method.

**TABLE 2:** Characteristics of Studies

References	Study Design	Diagnoses Methods	Study Period	Area	Continents
Meikle et al^10^	Retrospective case studies	Enzymatic assay	January 1, 1980-December 31, 1996	Australia	Oceania
Poorthuis et al^11^	Retrospective study	Enzymatic assay	1970-1996	Netherlands	Europe
Applegarth et al^12^	Unclear	Enzymatic assay/molecular analysis	1972-1996	British Columbia	North America
Dionisi-Vici et al^13^	Retrospective study	Enzymatic assay/molecular analysis	January 1, 1985-December 31, 1997	Italy	Europe
Asuman Ozkara and Topcu^14^	Records from a list of sources	Enzymatic assay	1997–2002	Turkey	Europe
Revest et al^15^	Retrospective study	Enzymatic assay/molecular analysis	Unclear	France	Europe
Poupetova et al^16^	Retrospective study	Enzymatic assay/molecular analysis	1975-2008	Czech Republic	Europe
Giraldo et al^17^	Retrospective study	Enzymatic assay	1976-2002	Iberian Peninsula	Europe
Mechtler et al^18^	Prospective nationwide screening	Enzymatic assay/molecular analysis	January 2010-July 2010	Austria	Europe
Stirnemann et al^19^	Retrospective study	Enzymatic assay	1980-2010	France	Europe
Liao et al^20^	Unclear	Enzymatic assay/molecular analysis	September 2011-January 2013	Taiwan	Asia
Hult et al^21^	Retrospective study	Enzymatic assay	1990-2009	Sweden	Europe
Hopkins et al^22^	Pilot study	Enzymatic assay/molecular analysis	January 11, 2013-June 11, 2013	Missouri	North America
Burton et al^23^	Unclear	Enzymatic assay/molecular analysis	November 1 2014-August 31 2016	Illinois	North America
Kang et al^24^	Unclear	Enzymatic assay/molecular analysis	Unclear	China	Asia
Hopkins et al^25^	Prospective study	Enzymatic assay/molecular analysis	January 11, 2013-January 10, 2017	Missouri	North America
Burlina et al^26^	Retrospective study	Enzymatic assay/molecular analysis	September 2015-January 2017	North East Italy	Europe
Chien et al^27^	Retrospective study	Enzymatic assay/molecular analysis	March 2018-April 2019	Taiwan	Asia

**FIGURE 2:**
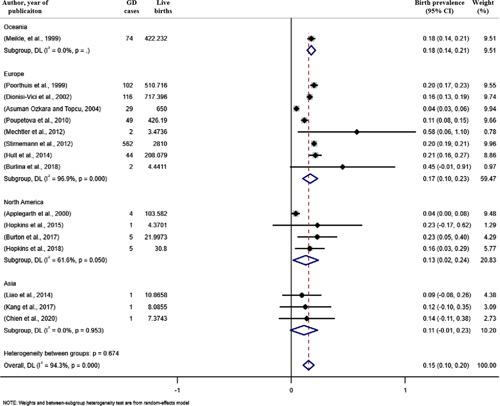
Forest plot of the estimated birth prevalence of Gaucher disease per 100,000 cases along with 95% confidence interval. Weights and between-subgroup heterogeneity test are from random-effects model.

Birth prevalence data of GD were extracted from 16 studies and covered 14 areas/countries. The global birth prevalence of GD was 1.5 cases (95% confidence interval [CI]: 1.0 to 2.0) per 100,000 live births. Birth prevalence of GD in Oceania, Europe, North America, and Asia were 1.8 cases [95% CI: 1.4 to 2.1], 1.7 cases [95% CI: 1.0 to 2.3], 1.3 cases [95% CI: 0.2 to 2.4], and 1.1 cases [95% CI: −0.1 to 2.3] per 100,000 live births, respectively (Fig. 2).

**FIGURE 3:**
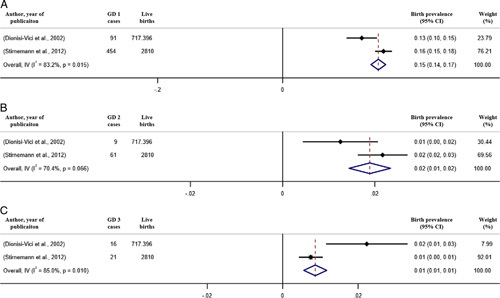
Forest plot of the estimated prevalence of Gaucher disease (GD) per 100,000 cases along with 95% confidence interval. Weights and between-subgroup heterogeneity test are from random-effects model.

Birth prevalence of GD type 1 [GD 1], type 2 [GD 2], and type 3 [GD 3] were 1.5 cases [95% CI: 1.4 to 1.7], 0.2 [95% CI: 0.1 to 0.2], and 0.1 [95% CI: 0.1 to 0.1] per 100,000 live births, respectively (Fig. 3).

**FIGURE 4:**
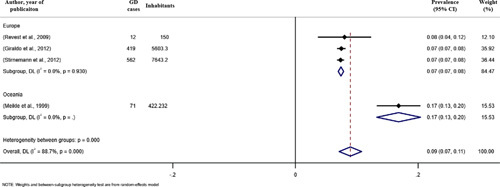
Forest plot of the estimated birth prevalence of Gaucher disease (GD) type 1 (A), type 2 (B), and type 3 (C) per 100,000 cases along with 95% confidence interval. Weights and between-subgroup heterogeneity test are from random-effects model.

Prevalence data of GD were extracted from 4 studies and covered 3 areas/countries. The global prevalence of GD was 0.9 cases [95% CI: 0.7 to 1.1] per 100,000 inhabitants. The prevalence of GD in Oceania and Europe were 1.7 cases [95% CI: 1.3 to 2.0] and 0.7 cases [95% CI: 0.7 to 0.8] per 100,000 inhabitants, respectively (Fig. 4).

**FIGURE 5:**
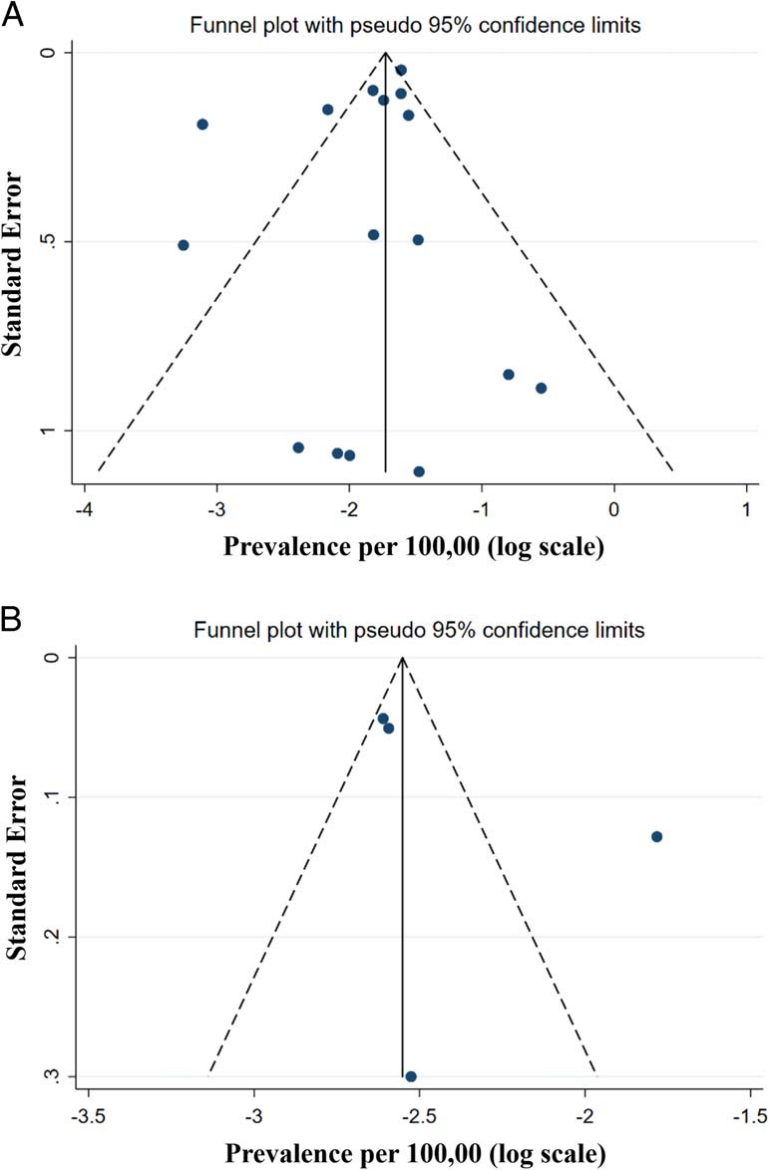
Funnel plot for the estimated birth prevalence (A) and prevalence (B) of Gaucher disease.

Although the range of birth prevalence and prevalence of GD was large, no qualitative difference in study methodology that could justify its impact on the pooled estimates was observed. No publication bias was found based on the funnel plot and Begg’s test for birth prevalence and prevalence of GD [*P* value=0.274 and 0.389; Fig. 5].

## DISCUSSION

The upper limit in the definition of rare disease ranges from 5 to 76 cases per 100,000 people.^28^ According to the definition of rare disease, quantitative epidemiology of GD in this study (1.5 cases [95% CI: 1.0 to 2.0] per 100,000 live births) confirmed that GD was a rare disease.^29^ GD is extremely common in Ashkenazi Jews. Goldblatt and Beighton^30^ reported that the prevalence of GD in the South African Jewish population would be 1:4000. Through population-based genetic screening programs, birth prevalence of GD was predicted to be 1:450.^31^ In Ashkenazi Jews, GD may not be considered as a rare disease; however, epidemiology data of GD in one race could not represent other races.

Because of the founder effect, the data from a specific population from one area/country may affect the accuracy of global epidemiology of GD, so 3 studies that focused on 1 specific population were excluded: (1) Goldblatt and Beighton^30^ reported prevalence of GD in Jewish population (1:4000); (2) Swart et al^32^ reported prevalence of GD in Cape Coloured population (1:247350); (3) Limgala et al^33^ screened 3 GD patients in 5287 people (~90% reported as African-American).

When studies were screening and quality assessing, “incidence” was misused to present the frequency of GD among births. It is easy to distinguish the difference between incidence and prevalence. The numerator and denominator of incidence are the numbers of disease onsets and the number of healthy individuals [a population at risk] during periods of observation. The numerator and denominator of prevalence are the total number of cases and the number of population at a certain moment.^34^ For new born screening of genetic diseases, including GD, patients were already there, so incidence is not suitable for frequency of GD among births. Birth prevalence, the prevalence at birth, was more suitable to present the frequency of GD among births.^8^,^35^

Theoretically, prevalence should be not far from birth prevalence.^36^ In this review, birth prevalence of GD (1.5 cases [95% CI: 1.0 to 2.0] per 100,000 live births) was higher than prevalence of GD (0.9 cases [95% CI: 0.7 to 1.1] per 100,000 inhabitants). Following reasons could explain such phenomenon: (1) it is very hard to find all GD patients; (2) the life span of GD patients is not long enough as a normal person.^37^ Although birth prevalence was affected by many factors, including diagnostic technology, prenatal diagnosis, and termination of pregnancy, birth prevalence of GD may be more accurate than prevalence to calculate the number of GD patients for now.

To our best knowledge, there was only one comprehensive review to represent GD epidemiology.^5^ In this review, the author used incidence and birth prevalence at the same time to show the frequency of GD among births, which would confuse the reader. In the part of “incidence”, 11 studies were used to review “incidence” of GD.^5^ Among these 11 studies, 8 studies were included in this review to calculate the birth prevalence of GD.^10^,^12^–^14^,^18^,^19^,^21^,^22^ In the part of “prevalence”, prevalence of GD was reviewed on the basis of 9 studies.^5^ Among these 9 studies, 3 and 2 studies were included in this review to calculate prevalence^10^,^19^,^38^ and birth prevalence of GD,^11^,^16^ respectively.

Pooled birth prevalence of GD in Europe was lower than the data in Oceania; however, the highest birth prevalence of GD was reported in Austria from Europe [5.8 cases per 100,000 live births].^18^ The lowest birth prevalence of GD in Europe, 0.2 cases per 100,000 live births, was reported in Turkey.^14^ The difference of GD birth prevalence between Austria and Turkey may be explained by the proportion of Ashkenazi Jews in the two countries.^30^,^31^ Three studies of GD birth prevalence in Asia were all from China, which has a low proportion of Ashkenazi Jews. If pooled birth prevalence of GD in Asia contained data from West Asia, the birth prevalence of GD in Asia may be higher.

According to pooled birth prevalence of three subtypes of GD, proportion of patients with GD 1 in total patients with GD is ~83%, which is consistent with the review of Stirnemann et al [prevalence of GD1: 90%–95% in Europe and North America].^1^ There are two other studies that reported birth prevalence of 3 subtypes of GD; however, cases of GD 1 patients were separated into 2 groups [early and late], meanwhile cases of GD 2 patients and GD 3 patients were mixed.^11^,^16^ These two studies were excluded.

Quantitative data of GD global epidemiology is the fundamental to evaluate global efforts that build a better world for GD patients, including more accurate data collection, development of diagnostic technology and new therapies. Life expectancy would be an example to clarify this point. Life expectancy has increased by more than 6 years between 2000 and 2019: from 66.8 years in 2000 to 73.4 years in 2019, globally.^39^ Life expectancy (from 66.8 years in 2000 to 73.4 years in 2019) of whole-world is pooled global data to show the global efforts to expand life expectancy of citizens worldwide. Global efforts, including the development of medical technology, food supply, and reduction of war, were carried out by governments around the world. Unfortunately, life expectancy varies broadly in different countries in 2019, from 50.75 years in Lesotho to 84.26 years in Japan.^40^ The broadly varied data of life expectancy did not reduce the effect that higher pooled global data could reflect global efforts to expand life expectancy of citizens worldwide.

### Limitations

There are several limitations of this report: (1) <30% of studies were assessed as high-quality, highlighting the need for high-quality study about epidemiological evidence of GD; (2) more than half of the studies were from Europe (56%). Reports from other continents were underrepresented, which might cause bias for calculating global epidemiology of GD.

## CONCLUSIONS

To our best knowledge, this is the first systematic review to present quantitative data of GD global epidemiology. Quantitative GD global epidemiology is the fundamental to evaluate the global efforts that build a better world for GD patients.
